# Opportunities and challenges of patient-derived models in cancer research: patient-derived xenografts, patient-derived organoid and patient-derived cells

**DOI:** 10.1186/s12957-022-02510-8

**Published:** 2022-02-17

**Authors:** Xiaoying Hou, Cong Du, Ligong Lu, Shengtao Yuan, Meixiao Zhan, Pengtao You, Hongzhi Du

**Affiliations:** 1 Wuhan Institutes of Biomedical Sciences, School of Medicine, Jianghan University, Wuhan, 430056 China; 2 Guangdong Provincial Key Laboratory of Liver Disease Research, The Third Affiliated Hospital, Sun Yat-sen University, Guangzhou, 510620 China; 3 Zhuhai Interventional Medical Center, Zhuhai Precision Medical Center, Zhuhai People’s Hospital, Zhuhai Hospital Affiliated with Jinan University, Zhuhai, 519000 China; 4 Jiangsu Key Laboratory of Drug Screening, Jiangsu Center for Pharmacodynamics Research and Evaluation, China Pharmaceutical University, Nanjing, 2100 9 China; 5 School of Pharmacy, Hubei University of Chinese Medicine, Wuhan, 430065 China

**Keywords:** Patient-derived model, Patient-derived xenograft (PDX), Patient-derived organoid (PDO), Patient-derived cell (PDC), NCI-60

## Abstract

**Background:**

As reported, preclinical animal models differ greatly from the human body. The evaluation model may be the colossal obstacle for scientific research and anticancer drug development. Therefore, it is essential to propose efficient evaluation systems similar to clinical practice for cancer research.

**Main body:**

While it has emerged for decades, the development of patient-derived xenografts, patient-derived organoid and patient-derived cell used to be limited. As the requirements for anticancer drug evaluation increases, patient-derived models developed rapidly recently, which is widely applied in basic research, drug development, and clinical application and achieved remarkable progress. However, there still lack systematic comparison and summarize reports for patient-derived models. In the current review, the development, applications, strengths, and challenges of patient-derived models in cancer research were characterized.

**Conclusion:**

Patient-derived models are an indispensable approach for cancer research and human health.

## Background

Cancer is one of the most major threats to human life and health worldwide [1]. The abundance of pharmaceutical companies have been dedicated to screening effective and novel anticancer drugs. However, the development of a single innovative anticancer drug always requires billions of dollars and decades of years [2, 3]. Meanwhile, when tested in clinical trials, only ~ 5% of the drug candidates can be approved by the governmental drug administration [2, 4]. According to the reports, limited efficacy accounted for more than 60% of failures during clinical trials in cancer therapy [5, 6]. While the therapeutic effect of each drug candidate is sufficiently confirmed through cell biology and animal models in preclinical studies, the heterogeneity between preclinical models and the human body leads to extensive clinical trial failure [7–9]. The evaluation model may be the colossal obstacle for scientific research and anticancer drug development.

NCI-60, a panel of 60 human cancer cell lines established by the US National Cancer Institute (NCI) in 1990, has been the fundamental tool in cancer research and widely applied in vitro and in vivo [10]. Despite it being derived from actual patients, NCI-60 cells were still much different from clinical practice [10]. First, the genetic composition and behavior of NCI-60 cells altered after thousands of generations in culture [10]. Importantly, heterogeneity and microenvironment are not homologous when NCI-60 cell lines are applied alone in research [11], which is recognized as a key factor for cancer development [12]. Existing studies suggested that various classical cancer cell lines have been polluted or mixed by others cells [13, 14], leading to inaccurate results based on NCI-60 cell line-derived models. Therefore, despite more than 25 years of extensive use by researchers, NCI decided to stop the application of NCI-60 for drug screening in 2016 [10]. As a result, it is urgent to propose efficient evaluation models similar to clinical practice in cancer research.

## Patient-derived models for cancer research

**Fig. 1:**
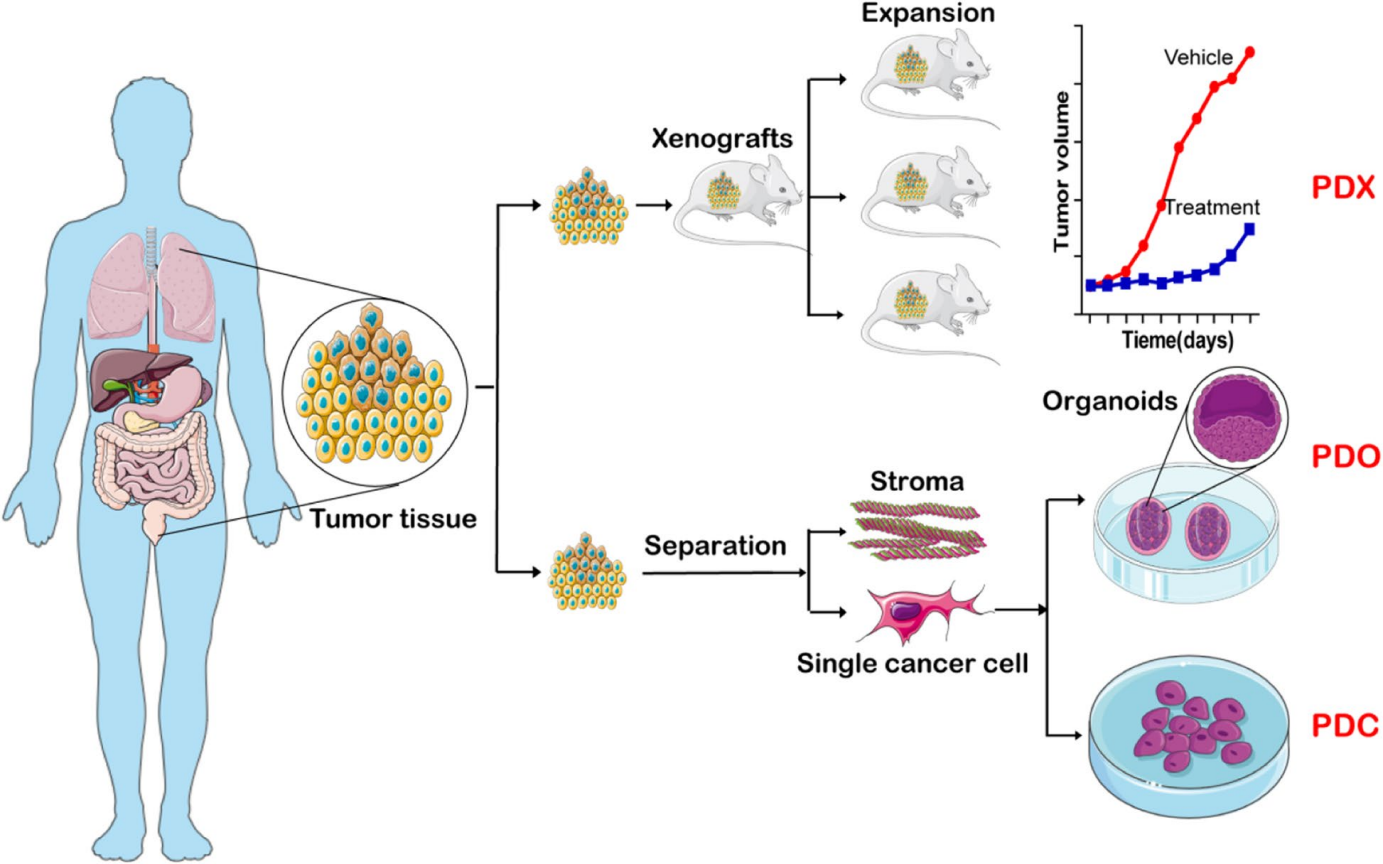
Schematic of three patient-derived models: PDX, PDO, and PDC. The process to establish the three patient-derived models: PDX, PDO, and PDC, respectively. All the three derived from the actual patient’s tumor tissue, but under different approaches

NCI-60–derived models have been the critical tools over the past 30 years, but a large number of researches proposed that NCI-60–derived models differ greatly from clinical cancer patients [9, 10]. In this context, the concept of patient-derived models emerged and gained extensive acceptance in cancer research. For the latest drug evaluation standards, only the result confirmed by patient-derived models is veritable, especially in translational medicine [7]. According to existing reports, there are three patient-derived models (Fig. 1) developed vigorously in the last 5 years: patient-derived xenografts (PDX), patient-derived organoid (PDO), and patient-derived cells (PDC).

### PDX

**Fig. 2:**
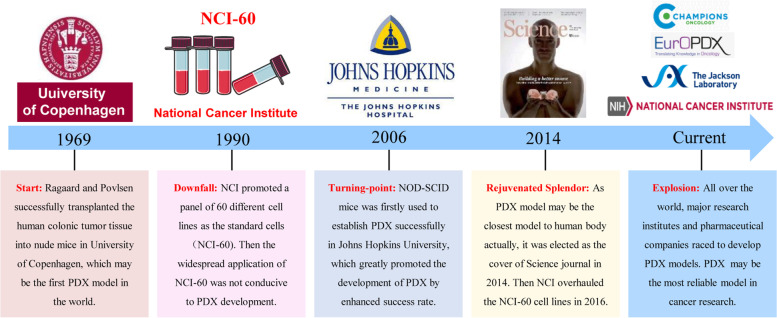
The development of the PDX model. Historical events underlying development of PDX model [15–18]

In fact, PDX was not the emerging technology in cancer research. In 1969, Ragaard and Povlsen successfully transplanted the human colonic tumor tissue into nude mice in Municipal Hospital, University of Copenhagen (Fig. 2) [15], this might be the first PDX model according to retrievable reports. However, the limited success rate of PDX modeling seriously hindered its development. Moreover, due to the characteristics of easy handling, rapid amplifying and yielded acceptable results, the application of NCI-60 cell lines remarkably increased in laboratories since 1990 [16]. Hence, the PDX model had been underdeveloped for many years. Until in 2006, Hidalgo et al. successfully established a PDX model through NOD-SCID (non-obese diabetes–severe combined immunodeficiency) mice at Johns Hopkins University [17]. Their study significantly facilitated the PDX development by enhancing the success rate of modeling. At the same time, a series of problems of NCI-60 cell lines were widely recognized by global researchers [19]. As a result, the PDX model gained popularity at the beginning of the 21st century. Increasing number of institutes, especially the pharmaceuticals companies, prefer to choose the PDX model in pharmacodynamics studies [20, 21]. In 2014, the PDX model was emphasized on the cover of *Science* journal for its close association with clinical practice [18]. NCI also declared that the NCI-60 cells would be retired in 2016 [10]. Therefore, major research institutes and pharmaceutical companies are racing to develop PDX models in recent years. PDX is recognized as the perfect model for anticancer drug evaluation.

### PDO

**Fig. 3:**
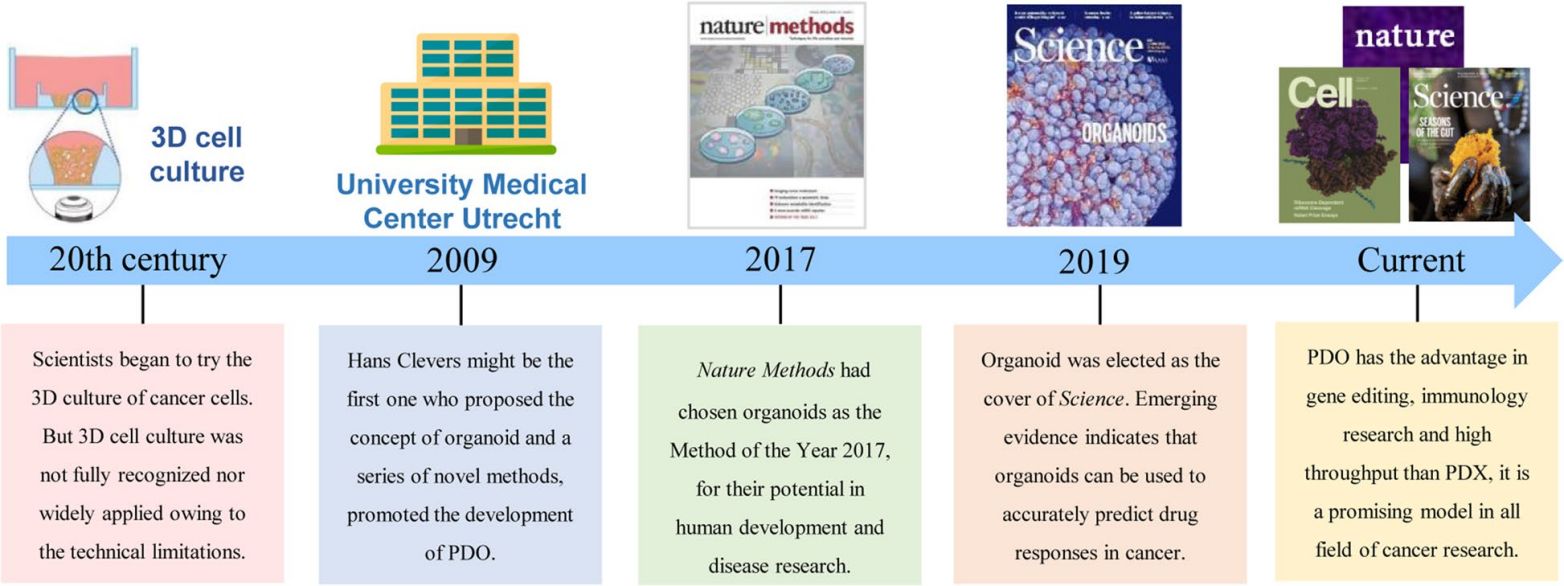
The development of the PDO model. Historical events underlying development of PDO model [25–28]

While it presumably provides the most reliable simulation of the human body, the PDX model still faced challenges such as unsatisfied success rate, limited throughput screening efficiency, high cost, and time-consuming nature [22, 23]. Thus, another drug evaluation model, PDO, also gained prominent development in the recent 5 years [24]. As shown in Fig. 1, patient-derived tumor tissue is firstly digested into single cells or clusters (diameter less than 100 μM) and transplanted to a basement membrane extract with specific growth medium. The organoid (patient-derived organoid) would be organized successfully in several weeks. As reported [25], the idea and trial of organoid cultured in vitro have been ongoing for a considerable period of time. Towards the end of the 20th century, scientists began the trial on the three-dimensional (3D) culture of cancer cells (Fig. 3) [26]. Compared with traditional two-dimensional (2D) cell culture, 3D cell culture was presumed to be more representative of human body [29]. Due to technical limitations, 3D cell culture used to be scarcely utilized. Until 2009, Hans Clevers proposed the definition of the organoid and demonstrated a series of novel methods for organoid culture [27], which greatly promoted the development of PDOs. With the groundbreaking discovery by Clevers, researchers performed similar studies in the following years. The organoid was even elected as the “Biotechnology of The Year” by *Nature Methods* in 2017 for its development and prospects [28]. Subsequently, journal of *Science* selected the significance of PDOs as the cover of 2019 [25]. Obviously, PDOs, also known as “organoid”, is an important preclinical evaluation model in cancer research.

### PDC

**Fig. 4:**
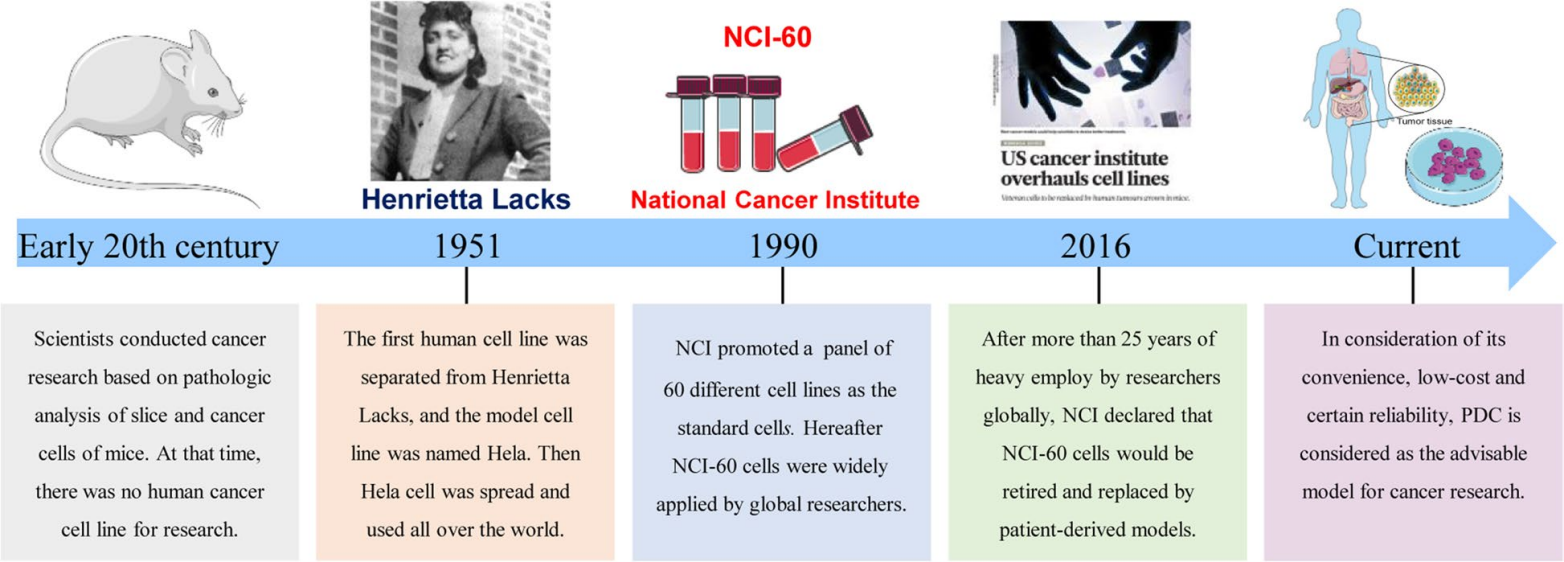
The development of the PDC model. Historical events underlying development of PDC model [30, 31]

Based on existing studies, PDXs and PDOs are supposed to be the most homologous in simulating human body and disease. However, both the two models are expensive and time-consuming, leading to limited utilization rate in cancer research. Meanwhile, studies suggested that NCI-60 cell lines are discontented due to the genetic and behavior alteration after thousands of generations. Classical cancer cell lines is no longer a credible model for their contamination by other cells or mycoplasma [13, 14]. As a result, the PDC model emerged as an ideal substitute for traditional cancer cell lines [30]. As is shown in Fig. 4, there used to be no human cancer cell line available in the early 20th century. In 1951, the first human cell line was separated from an American cancer patient named Henrietta Lacks, which is the first PDC model in the world [31]. Since then, countless PDCs were established by institutions and became an important research model. However, in 1990, NCI proposed a panel of 60 different cell lines as the standards to assist scientists conducting research concisely and normatively [16], which were subsequently widely applied. During the heavy use for 25 years, increasing number of reports [13, 14] suggested that NCI-60 exhibited gene mutations, biological function changes, and even pollution. Therefore, PDCs got renewed interest from researchers in consideration of its convenience, low cost, and certain reliability. Up to now, PDCs have been an indispensable approach for cancer research, especially in the early stages of research studies.

## Application

**Fig. 5:**
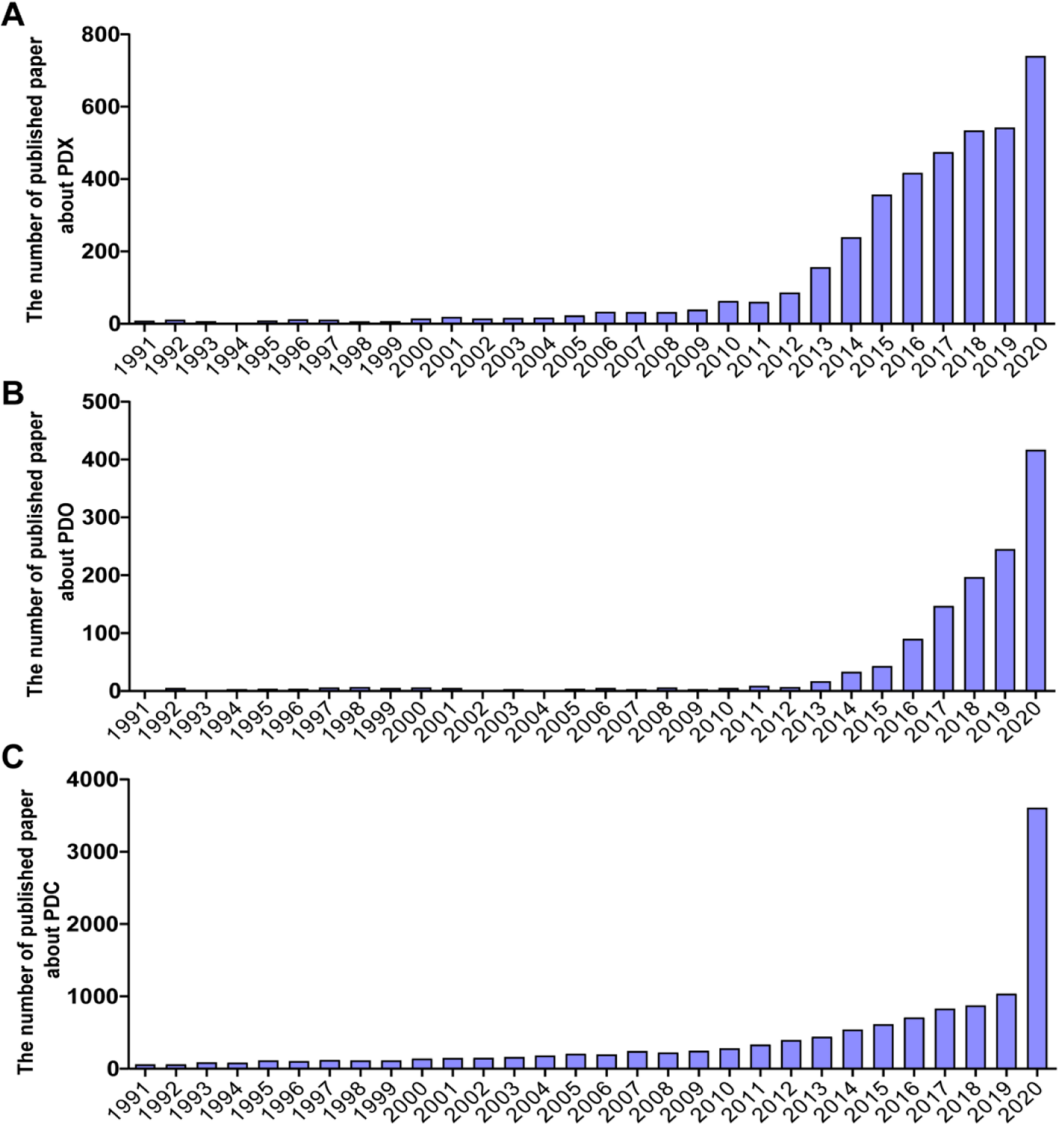
Popularity of the three patient-derived models in cancer research. The number of published papers about three patient-derived models is based on Web of Science, respectively. **A** Topic = (TOPIC: (Patient-Derived Xenograft) AND TOPIC: (Cancer). **B** Topic = ((TOPIC: (Patient-Derived Organoid) OR TOPIC: (Organoid)) AND TOPIC: (Cancer). **C** Topic = (TOPIC: (Patient Derived cell) AND TOPIC: (Cancer)

**Fig. 6:**
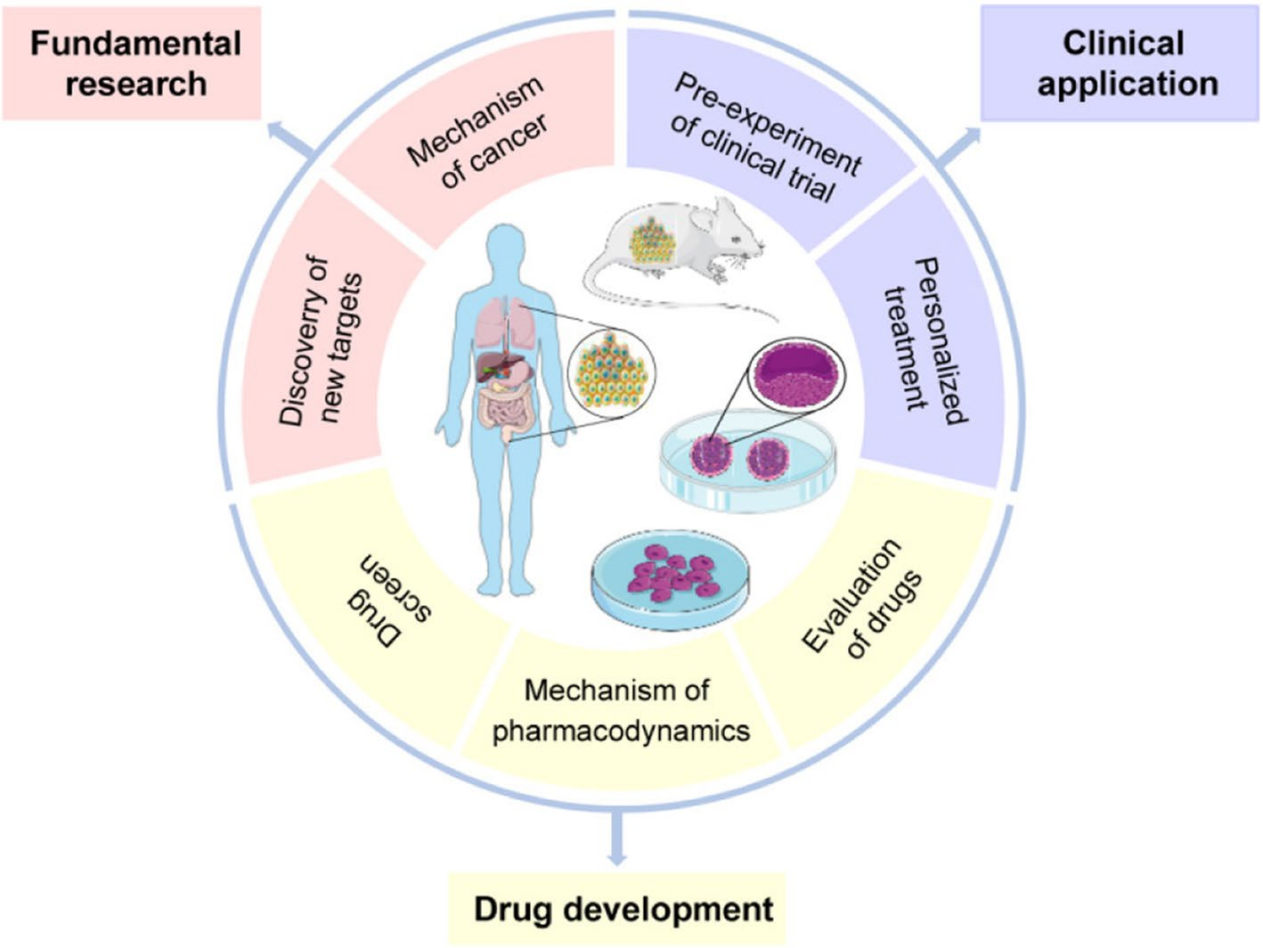
Applications of patient-derived models in cancer research. The patient-derived models (PDX, PDO, and PDC) can be applied in multi-fields of cancer research: fundamental research, drug development, and clinical application

While initially established decades ago, the development of PDX, PDO, and PDC models used to be restricted due to biotechnological limitations. With the progress in science and technology, these patient-derived models were gradually recognized and vigorously developed in cancer research (Fig. 5). The utilization of these models significantly increased around the world especially in the recent 5 years. At present, patient-derived models are widely applied in various areas of medicine including fundamental research, drug development, and clinical applications (Fig. 6). Apparently, the three patient-derived models will contribute to the anticancer development in the future.

### Fundamental research

Instead of traditional NCI-60–derived models, patient-derived models were recently used to verify the results concluded by traditional cell line–derived models. For instance, research institutions confirmed that feedback activation of leukemia inhibitory factor receptor is responsible for the limited efficacy of histone deacetylase inhibitors in breast cancer via the PDX model [32]. Meanwhile, superior to traditional cell lines, PDX could simulate the tumor microenvironment of actual patients [33], which is employed to reevaluate the cancer biomarkers screened by traditional technologies [34]. The PDO model is widely applied in fundamental cancer research as well. Compared with PDX, PDO is genetically editable, which is beneficial for investigating the mechanism of tumorigenesis [35] and tumor immune escape [36]. In addition, as an optimal substitution of NCI-60–derived models, PDC is also extensively used in fundamental cancer research [37].

### Drug development

Because of the advantages such as easy manipulation and rapid amplification, NCI-60 is still widely used in global laboratories, especially for the fundamental research laboratory [38, 39]. While the repeatability has been approved in vivo, conclusions based on NCI-60 could not readily translate to the human body [7]. Translational medicine institutions and pharmaceutical companies are no longer satisfied with the pharmacodynamics result from NCI-60–derived models alone. The large gap between preclinical evaluation models and clinical practice greatly promoted the adoption of new technologies, including the three patient-derived models described above.

Above all, the PDX model is regarded as the pre-experiment of clinical trial phase II and sometimes called “clinical trial phase 0” [40]. Before approval in clinical trials, the efficacy of innovative anticancer drugs such as Osimertinib [41], Nivolumab [42], and Bortezomib [43] had been evaluated by PDX. Besides, PDX is also an appropriate tool to discover drug-sensitive markers and screen drug combination strategies. Similarly, the role of PDO has already been confirmed in the evaluation of Nivolumab [44], Ganetespib [45], Proanthocyanidins [46], etc.. PDO may be the ideal model for high-throughput screening of drugs [36, 47]. Finally, the PDC model, an optimal substitute of NCI-60, is widely used for anticancer drug discovery and development, particularly in early stages of drug screening [48]. In a word, PDX, PDO, and PDC are indispensable models in drug development.

### Clinical application

Inherent heterogeneity of cancer patients always leads to dramatically different response to the same treatment. Thus, personalized therapeutic strategy, also called precise treatment or personalized treatment, is required in clinical practice [49]. However, it is impractical to evaluate the hundreds of drug candidates independently and annotate the ideal strategy. Fortunately, patient-derived models emerged as a substitute of patient to identify the optimal therapeutic strategy. Directly derived from the cancer patient, PDX may be the most homologous model of the human body which can be used to screen effective therapeutic strategy for a specific patient in the clinic. According to existing reports, the overall predictive accuracy of PDX was 90%, indicating that PDXs were optimal substitutes of the patients themselves [20, 50]. Nowadays, certain hospitals and research institutions have already provided personalized therapeutic regimen for their patients. While only highly malignant tumor is achievable to establish a PDX model [22, 23], PDO could be the replacement for low-grade tumors [51]. Meanwhile, PDO is used to identify effective regime for cancer patients [52], with the predictive accuracy more than 80% [53]. Additionally, due to the time-consuming and costly nature of PDX and PDO, the PDC model is always selected in the preliminary screen of personalized treatment [50]. Directly derived from the patient, PDC can absolutely provide reliable positive predictions. In China, institutions also developed and implemented novel technologies known as mini-PDX. In brief, patient-derived models are important substitutes of patients for annotating effective personalized treatment in clinical.

## Strengths and challenges

Although PDX, PDO, and PDC are not emerging models, they have been rejuvenating over the past 10 years with considerable success. Derived from actual patients, the results obtained from them are more factual, reliable, and effective. Each of the patient-derived models has their own advantages. An increasing number of studies chose patient-derived models for drug evaluation and biomarker annotation (Fig. 6**)**. However, there remained several obstacles limiting their continued development.

### Strengths and challenges of the PDX model

While all derived from patients, typically, the PDX model alone is implemented in vivo, which is known to be more accurate and reliable compared with that in vitro. As is acknowledged, PDX may be the ideal model to simulate actual human disease for cancer research thus far [54]. In research institutions, pharmaceutical companies, and medical organizations, the PDX model is widely applied in fundamental research, drug development, as well as clinical practice. However, there remain some challenges. First, the establishment of a PDX model often requires several months and considerable expenditures, which is fatal for researches and patients [23, 50]. Second, the success rate is unsatisfactory, and only highly malignant tumors are applicable for PDX [50]; a few cancer patients could benefit from it. Third, the application of PDX requires strict and time-consuming ethical approval process. Fourth, the intrinsic genetic material and cellular characteristics of tumor tissue will be altered after three generations, the PDX model is not suitable for continuous amplification [55]. In addition, immune deficiency is indispensable for the PDX model, it is difficult to conduct studies on cancer immunity [56]. Fortunately, humanized mice and mice with reconstituted human immune systems are under investigation [57]. In conclusion, all these challenges indicate that PDX is hard to popularize currently, but it will be widely utilized with the progress of science and technology in the future.

### Strengths and challenges of the PDO model

Compared with PDX and PDC, the PDO is the frontier of science and technology. According to the previous publications, while it produces accurate simulations of human disease [51], PDO shows the advantages for editable genes [36] and is applicable for immunity investigations [35], compensating the limitations of the PDX model. Superior to the PDX, tumors in all grades can be theoretically used to establish a PDO [51]. Currently, PDO is commonly utilized in high-throughput screening, indicating the potential role of it in drug development [36, 58]. However, establishment of a PDO model is also time-consuming, costly, and technically difficult. Therefore, further exploration on PDOs is still required.

### Strengths and challenges of the PDC model

In contrast to PDX and PDO, the PDC model is more convenient and economical, making it more popular and applicable in fundamental research and even in the screen of drug candidates [48, 50]. However, similar to NCI-60 cell lines, the source and quality of a PDC model can hardly be controlled. It is difficult to reproduce the experimental results among different PDC models. Moreover, the difference between PDC models and human body also affects the authenticity of research data [59]. Nevertheless, PDC is still a universal and fundamental patient-derived model for cancer research.

## Conclusions

In conclusion, the PDX, PDO, and PDC models are optimal humanized models currently in cancer research, which can successfully simulate the human body and clinical practice. The universalization of patient-derived models will support basic cancer research and provide additional scientific evidence for novel and effective drug development. For eventual clinical application, they will also yield more precise and reliable treatments. In contrast, with the improved importance of patient-derived models in cancer research, NCI-60–derived models will be gradually replaced in the future.

Nowadays, innovative technologies emerged to overcome the challenges and obstacles which limit the development of patient-derived models. For example, the success rate of the PDX model could be improved by the application of severe combined immunodeficiency mice such as NOG (NOD/Shi-scid/IL-2Rγ^null^), NCG (NOD-*Prkdc*^em26^*Il2rg*^em26^Nju), and NSG (NOD.Cg-*Prkdc*^scid^ *Il2rg*^tm1Wjl^/SzJ) [60, 61]. Humanized mice would be the optimized substitute of PDX in cancer immunology research [62]. Meanwhile, with the development of technologies, PDOs will become universal and available for more researchers [63]. In addition, a perfect system and stable source also promote the replacement of traditional NCI-derived models by PDC. In a word, patient-derived models have a bright prospect in cancer research but still is faced with several obstacles.

Notably, the PDX, PDO, and PDC models are not scientific breakthroughs. Various obstacles also existed which limit the development of patient-derived models. While it showed prominent advantages, there remains an urgent need for further development of these models. In brief, additional investigation will allow patient-derived models to become indispensable tools for cancer research and facilitate substantial contributions to human health in the future.

In a word, the current review systematically introduced the applications of patient-derived models (PDX, PDO, and PDC) in basic research, drug development, and clinical application, suggesting the inspiring insights on the strengths and challenges of the three models and providing a comprehensive evaluation system for cancer research.

## Data Availability

All data and materials used in this work will be available on request.
